# Multimodal MRI-based radiomics model for predicting short-term efficacy in nasopharyngeal carcinoma

**DOI:** 10.3389/fmed.2025.1654023

**Published:** 2025-11-21

**Authors:** Fei-yi Zhuang, Tian-xiu Zheng, Yi Wang, Ya-hong Li, Ping-zhen Chen, Bao-ling Li, Fei-lin Le, En-hui Qiu, Wei-yang Xu, Zhu-jian Chen, Xiao-fang Chen, Yuan-zhe Li

**Affiliations:** 1 Department of Otolaryngology, The Second Affiliated Hospital of Fujian Medical University, Quanzhou, Fujian, China; 2 Department of Radiology, Ningde Municipal Hospital of Ningde Normal University, Ningde, Fujian, China; 3 Department of CT/MRI, The Second Affiliated Hospital of Fujian Medical University, Quanzhou, Fujian, China

**Keywords:** nasopharyngeal carcinoma, MRI radiomics, treatment response prediction, support vector machine, multimodal imaging, clinical decision support

## Abstract

**Problem:**

Accurate prediction of short-term treatment response remains a critical challenge in nasopharyngeal carcinoma (NPC) management. Traditional TNM staging and clinical biomarkers offer limited precision for individualized therapy planning, creating a need for more robust, non-invasive predictive tools.

**Aim:**

This multicenter study aimed to develop and validate a multimodal MRI-based radiomics model for predicting short-term treatment response in NPC, and to compare its performance against conventional clinical biomarkers.

**Methods:**

We analyzed pre-treatment T1-weighted, T2-weighted, and contrast-enhanced T1-weighted MRI sequences from 173 patients in our primary cohort and 55 external validation cases. A total of 3,591 radiomic features were extracted per patient. After rigorous feature selection using maximum relevance minimum redundancy (mRMR) and Least Absolute Shrinkage and Selection Operator (LASSO) regression, we developed and compared eight machine learning classifiers. Model performance was evaluated through comprehensive validation, including calibration analysis and decision curve assessment.

**Results:**

The Support Vector Machine (SVM) model demonstrated superior performance, achieving an area under the curve (AUC) of 0.935 (95% CI: 0.867–1.000) on internal testing with balanced sensitivity (87.1%) and specificity (95.2%). External validation confirmed model robustness (AUC 0.880, 95% CI: 0.800–0.960). Our radiomics approach significantly outperformed all clinical biomarkers (AUC improvement: 18.7–24.3%, *p* < 0.01) and demonstrated clinical utility across decision probability thresholds of 12–48%.

**Conclusion:**

The multimodal MRI-based radiomics model represents a transformative non-invasive tool for predicting short-term treatment response in NPC, offering superior performance to conventional methods and providing valuable insights for personalized treatment strategies. Our findings support the integration of radiomics into clinical decision-making for NPC management.

## 1 Introduction

The NPC is a malignant tumor that develops from the mucosal lining of the nasopharynx. The main pathological type is non-keratinized squamous cell carcinoma, accounting for about 95% (1). It is commonly seen in Southeast Asian countries (2). Due to its deep anatomical location, high rate of lymph node metastasis, and aggressive invasion, NPC is often diagnosed at an advanced stage (3). Currently, oncologists mainly choose the appropriate treatment methods based on the TNM stage of the tumor. However, even if the patients with the same stage receive the same treatment, there will be different results (4). In the early stage, the main treatment method is radiotherapy, and for advanced patients, it is the combination of radiotherapy and chemotherapy. Compared with other malignant tumors, the prognosis of NPC is better, and the 5-year survival rate can be as high as 80% (5), but there are still some patients, after the end of treatment, the early tumor progression, metastasis, resulting in adverse consequences (6). Tumor TNM stage only reflects the outward invasion of the tumor but cannot evaluate the interior of the tumor. Many previous clinical studies have primarily concentrated on markers like Epstein-Barr virus (EBV) DNA antibody level, white blood cells, and neutrophil number, which are correlated with prognosis, but lack specificity (7).

Tumor progression and response to therapy are influenced by multiple factors that TNM staging alone cannot encompass, such as molecular and cellular characteristics within the tumor microenvironment. Recent studies have explored biomarkers, such as EBV-DNA levels, neutrophil-to-lymphocyte ratio, and lactate dehydrogenase (LDH) levels, as prognostic indicators in NPC. However, these markers often lack specificity and do not fully capture the tumor’s complexity (8).

MRI is essential for diagnosing, staging, and planning treatment for NPC. Compared with other imaging modalities, MRI offers excellent soft-tissue contrast, enabling detailed visualization of tumor boundaries, invasion into adjacent structures, and lymph node involvement (9). In MRI for NPC, each sequence provides unique diagnostic advantages. T1WI offers high anatomical detail with clear contrast between fat and fluids, essential for defining anatomical boundaries and accurately localizing the primary tumor. T2WI enhances fluid contrast, making it particularly effective for identifying inflammation, edema, and potential invasion into adjacent tissues, which indicates tumor extent and aggressiveness. contrast-enhanced T1-weighted, following gadolinium administration, highlights vascularized areas and tumor margins, enhancing lymph node visibility and aiding in precise staging of NPC. MRI’s high-resolution imaging capabilities are essential for accurately assessing the extent of NPC and monitoring treatment response, particularly in cases with complex anatomical relationships in the nasopharyngeal area. Moreover, MRI has the advantage of non-invasive, radiation-free, and is suitable for repeated evaluation of tumor therapy (9). Recent studies have advanced the computational analysis of NPC, including a trainable model for tumor segmentation (10), a review of electronic diagnostic methods (11), and a comprehensive survey of NPC research concepts and methodologies (12). While these contributions provide valuable foundations in segmentation and diagnostic technologies, they leave unmet needs in multi-institutionally validated, multimodal MRI-based predictive modeling for treatment response. Importantly, prior works seldom benchmark against clinical biomarkers or address model interpretability—key barriers to clinical adoption. Our study directly addresses these gaps by developing a rigorously validated, multimodal radiomics model evaluated against clinical standards and explained via SHAP analysis to support clinical decision-making. While several previous studies have explored radiomics for NPC prognosis (13, 14), significant limitations remain. First, many existing models derive from single-center cohorts without external validation, raising concerns about generalizability. Second, most approaches utilize single-sequence MRI data, potentially underutilizing the complementary prognostic information available across multiple sequences. Third, the clinical added value of radiomics models beyond established biomarkers like EBV DNA has not been comprehensively evaluated. Finally, there is often a lack of robust calibration and interpretability analysis, which are critical for clinical translation.

To address these gaps, we developed and validated a multimodal MRI-based radiomics model across multiple centers. Our approach specifically integrates T1, T2, and contrast-enhanced T1-weighted sequences to capture a more comprehensive range of tumor characteristics. We implemented stringent external validation using data from a completely separate institution to rigorously test generalizability. Furthermore, we directly compared our model against a comprehensive panel of clinical biomarkers and conducted detailed calibration and decision curve analysis to evaluate its clinical utility. Finally, we applied SHAP analysis to enhance model interpretability. This comprehensive approach aims to provide a robust, clinically applicable tool for personalized treatment planning in NPC.

Tumor development is a multifactorial process, and sufficient tumor data information contributes to tumor knowledge (15). With the advancements in artificial intelligence technology, Radiomics is a hot field at present (16). It has clinical significance and advantages for the extraction of internal tumor characteristics (17). The value of efficacy prediction and risk stratification of NPC based on imaging omics has been affirmed (18).

Most existing radiomics models for NPC are derived from single-center studies and lack rigorous external validation, raising significant concerns about their generalizability to unseen populations and different imaging protocols. The potential synergistic predictive value of combining quantitative features from multiple MRI sequences (T1, T2, CE-T1) within a unified model has not been fully explored, often relying on single sequences. The comparative performance of radiomics models against a comprehensive panel of standard clinical biomarkers (e.g., EBV DNA, LDH) is frequently not assessed, creating ambiguity regarding their actual added value in clinical decision-making. The “black-box” nature of many complex models hinders clinical adoption, as the underlying decision-making process and the most influential image features driving predictions are not revealed.

This study aims to develop a robust and interpretable model for predicting short-term treatment response in NPC using multimodal MRI-based radiomics (19). Our approach makes five key contributions: (1) the development of a multimodal MRI-based radiomics model integrating T1, T2, and contrast-enhanced T1-weighted sequences; (2) rigorous multicenter validation across independent cohorts to ensure generalizability; (3) comprehensive benchmarking against standard clinical biomarkers to quantify added value; (4) enhanced model interpretability through SHAP analysis to reveal decision-making processes; and (5) advanced performance evaluation using calibration metrics and decision curve analysis. By addressing these aspects, our model provides clinicians with a transparent and reliable tool for individualized treatment planning prior to therapy initiation (20, 21).

## 2 Patients and methods

**FIGURE 1:**
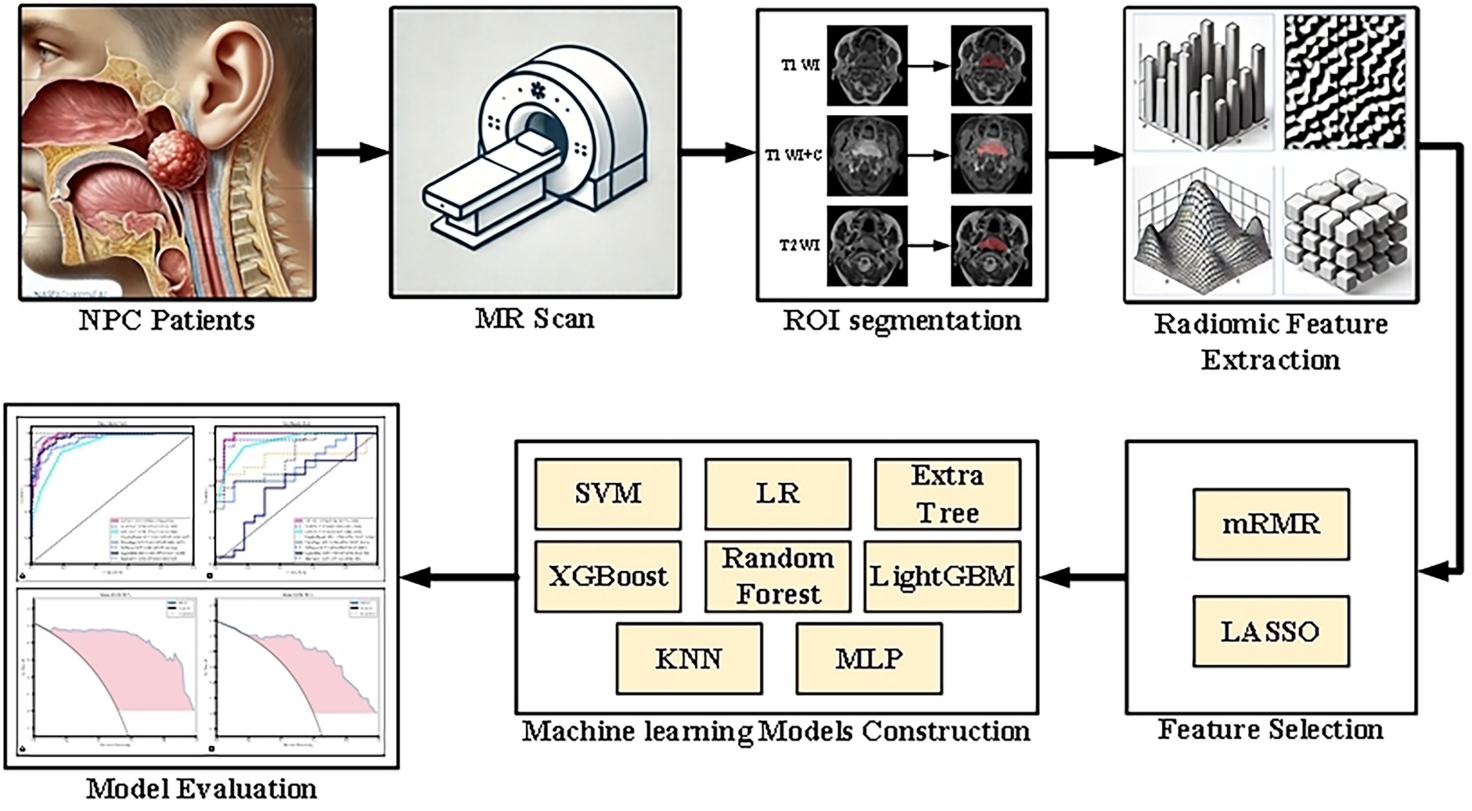
Flowchart of the research process, detailing image collection, ROI segmentation, radiomic feature extraction and selection, model construction, and performance evaluation.

The research process, as illustrated in Figure 1, begins with the acquisition of MRI images from NPC patients, followed by manual regions of interest segmentation (ROI). Radiomic features are then extracted from these images and undergo a feature selection process. Subsequently, multiple machine learning models are constructed and evaluated to determine the optimal model for predicting treatment outcomes.

### 2.1 Patients

**FIGURE 2:**
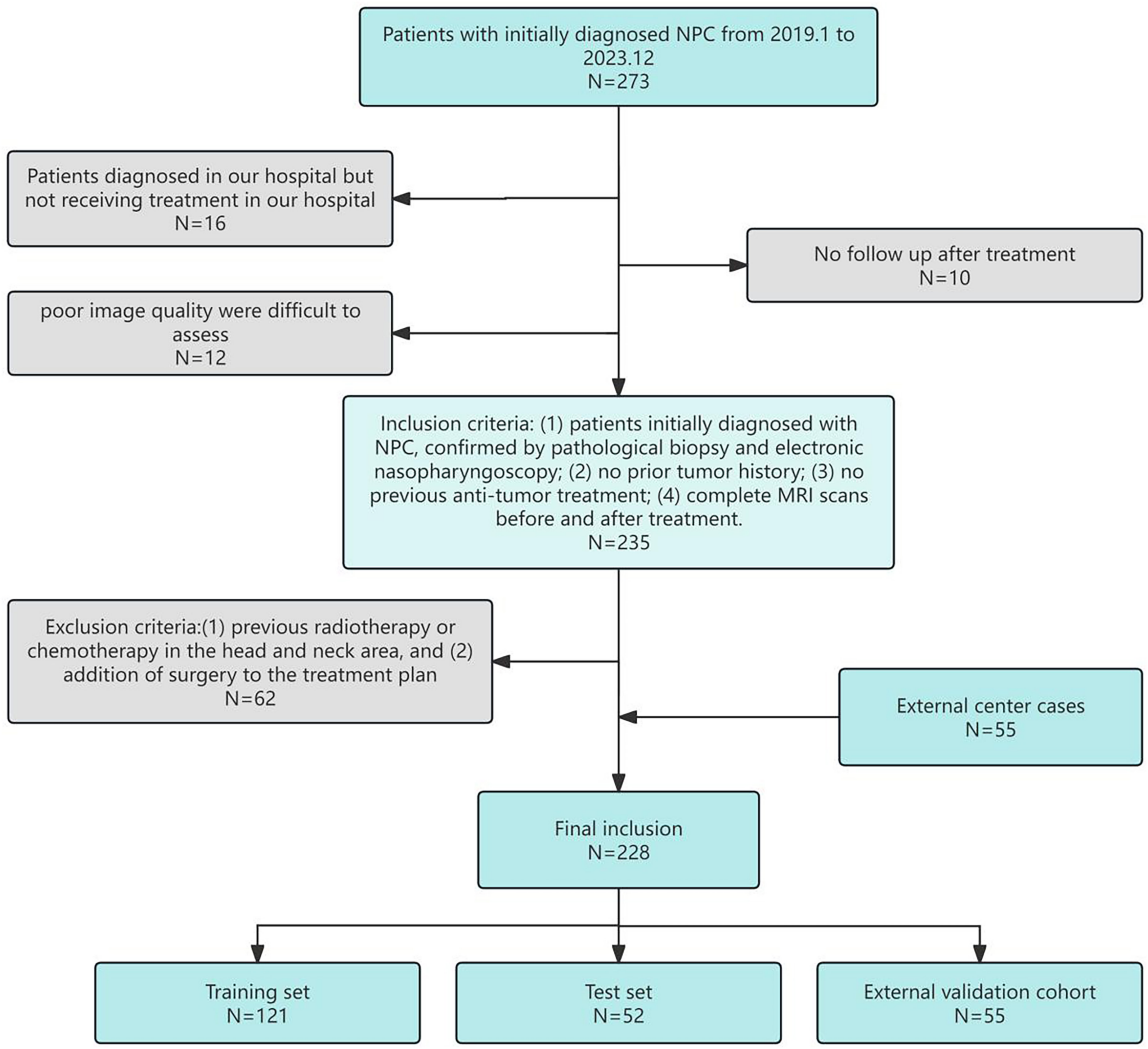
Patience selection flowchart.

This study analyzed data from 173 NPC patients (93 positive and 80 negative cases*) initially diagnosed at the Second Affiliated Hospital of Fujian Medical University between January 2019 and December 2023. Positive cases were defined as those showing complete metabolic response on post-treatment PET-CT, while negative cases had residual metabolic activity. To enhance the validity of our findings, we incorporated an additional 55 cases (37 positive and 18 negative) from Ningde Municipal Hospital as an external validation cohort during the same study period. Collected data included gender, age, clinical TNM stage (the American Joint Committee on Cancer Manual’s 8th edition served as the standard), treatment methods, and MRI before and after treatment. Patients received follow-up examinations every 3 months after treatment, primarily with MRI, for a minimum of 6 months. The study adhered to the Declaration of Helsinki and was approved by the Ethics Committee of the Second Affiliated Hospital of Fujian Medical University, with informed consent obtained from all participants. The patient selection process is detailed in Figure 2, the inclusion criteria included: (1) patients initially diagnosed with NPC, confirmed by pathological biopsy and electronic nasopharyngoscopy; (2) no prior tumor history; (3) no previous anti-tumor treatment; (4) complete MRI scans before and after treatment. Exclusion criteria included: (1) previous radiotherapy or chemotherapy in the head and neck area, and (2) addition of surgery to the treatment plan.

### 2.2 Treatment

Radical radiotherapy was used as the basic therapy. In radiotherapy, the tumor target area, covering the primary site and involved lymph nodes, the total dose was 66–70 Gy, 32–35 planned doses: 1 time/day, 5 days/week. The synchronous chemotherapy uses single-agent cisplatin. Induction chemotherapy was based on cisplatin and gemcitabine. Induction and concurrent chemotherapy were administered every 3 weeks.

### 2.3 Criteria for tumor response

Tumor response evaluation followed the Response Evaluation Criteria in Solid Tumors (RECIST 1.1) (22). Using MRI images taken before treatment and 6 months post-treatment, the radiologist delineated the maximum diameter at the maximum level of the tumor in different sequential images, calculated and finally averaged the tumor response. Clinical response outcomes 6 months after treatment were categorized as complete response, partial response, stable disease, and progressive disease. Patients with a complete response were grouped into the complete remission (CR) group, while the rest were classified as the non-complete remission (non-CR) group.

### 2.4 Collection of clinical data

Pre-treatment clinical data were gathered from the health information system of the Second Affiliated Hospital of Fujian Medical University. Fourteen key features were selected for analysis, encompassing patient demographics and laboratory values. These included age, gender, Ki-67 index, EBV-DNA levels, albumin (ALB), total cholesterol (TC), LDH, leukocyte count (WBC), thymidine kinase 1(TK1), lymphocyte count (LYM), D-dimer (D-D), neutrophil count (NEUT), T stage, N stage, and clinical tumor stage. These variables were analyzed to evaluate their associations with NPC prognosis and treatment response. Statistical analysis of clinical variables was performed in two stages. First, univariate logistic regression analyses were conducted for each of the fourteen pre-treatment clinical variables to evaluate their individual associations with treatment response (complete remission vs. non-complete remission). Continuous variables were presented as mean ± standard deviation and analyzed using the independent Student’s *t*-test or Mann-Whitney U test, as appropriate. Categorical variables were presented as frequencies and analyzed using the Chi-square test or Fisherical vat test. Subsequently, variables with a univariate significance level of *p* < 0.1 were entered into a multivariate logistic regression model using a stepwise selection method (both forward and backward) to identify independent predictors. A two-sided *p*-value < 0.05 was considered statistically significant in the final multivariate model. All statistical analyses were performed using Python (version 3.9) with the scipy and statsmodels libraries.

### 2.5 MRI data acquisition

All patients received imaging on a 3.0 Tesla (T) MRI scanner, which provides enhanced image resolution and contrast, crucial for detailed radiomic analysis in NPC. Three specific sequences were selected: T1WI, T2WI, and contrast-enhanced T1-weighted, each contributing distinct information about tumor characteristics. specific imaging parameters were set for T1WI, T2WI, and contrast-enhanced T1-weighted to capture high-resolution images for NPC evaluation. For T1WI, the repetition time (TR) was set to 400–700 ms, echo time (TE) to 10–20 ms, flip angle to 70–90°, slice thickness to 3–4 mm, field of view (FOV) to 220–250 mm, and matrix size to 256 × 256 or greater. T2WI parameters included a TR of 3,000–5,000 ms, TE of 80–120 ms, and a 90° flip angle, with the same slice thickness, FOV, and matrix size as T1WI. The contrast-enhanced T1-weighted sequence, obtained after intravenous injection of a gadolinium-based contrast agent, used a TR of 500–700 ms, TE of 10–20 ms, and a flip angle of 70–90°. Each sequence had a voxel size of approximately 1 × 1 × 3 mm, with an acquisition time of 3–5 min per sequence, depending on patient tolerance. These settings provided high-resolution, high-contrast images across the different sequences to facilitate precise tumor identification and analysis. To mitigate the potential effects of inter-scanner variability (domain shift) in this multi-center study, a standardized image preprocessing pipeline was implemented prior to radiomic feature extraction. All MRI volumes first underwent N4 bias field correction using SimpleITK to reduce intensity inhomogeneity (23). Subsequently, each image was resampled to an isotropic voxel size of 1.0 × 1.0 × 1.0 mm^3^ using B-spline interpolation to ensure spatial consistency across different scanners and protocols (24). Finally, Z-score normalization was applied independently to each sequence (T1, T2, and CE-T1) across the entire cohort, using the mean and standard deviation derived from all voxels within all segmented tumor volumes for the respective sequence, thereby standardizing intensity distributions (25). This comprehensive harmonization approach effectively minimized domain shift, as reflected in the limited performance degradation observed during external validation (inter-center AUC variance: 2.7%). Although advanced methods like ComBat harmonization remain valuable for larger cohorts, the current pipeline provided robust and practical standardization for the scale of this study (26).

### 2.6 Feature extraction and modeling

**FIGURE 3:**
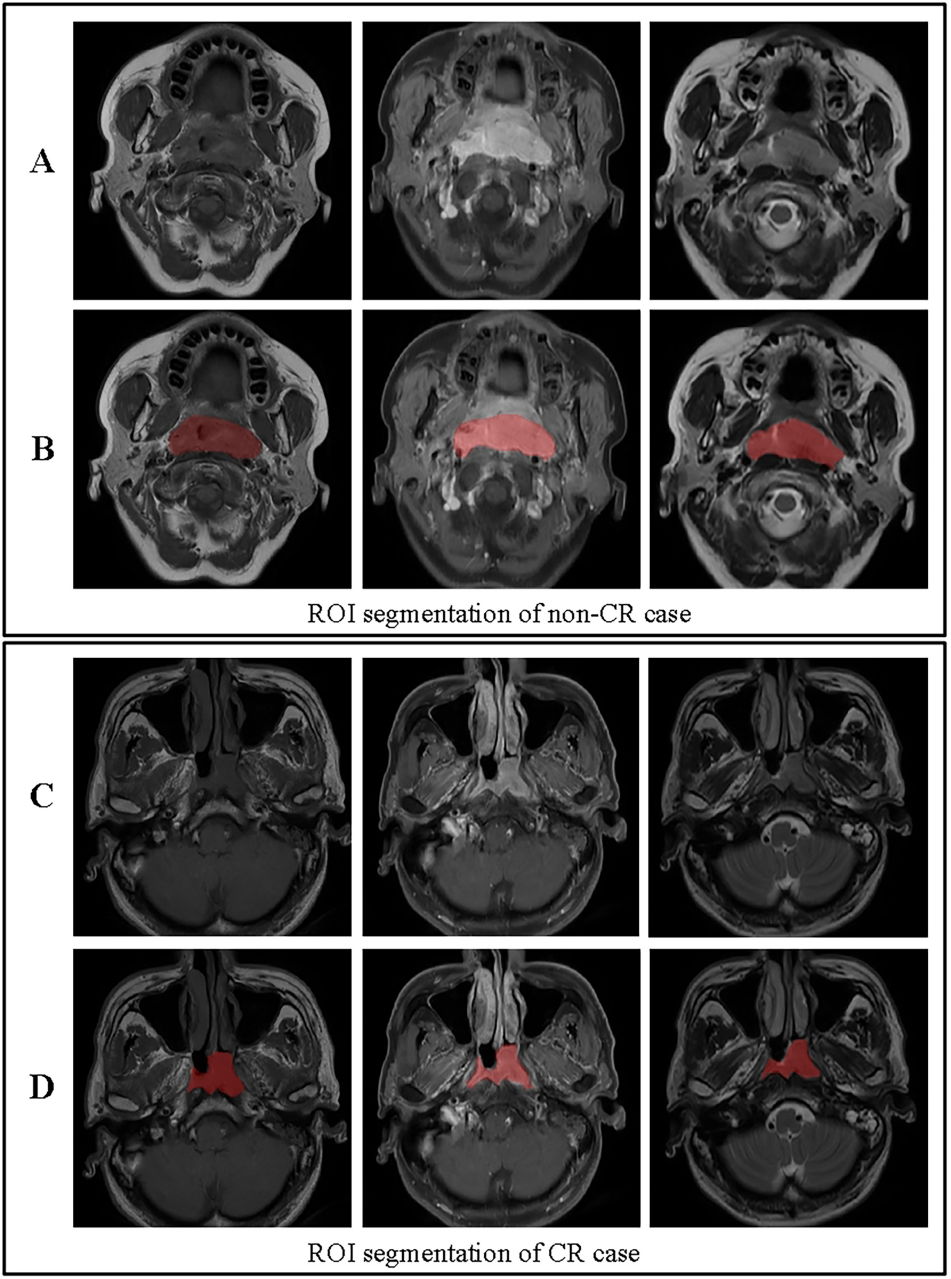
ROI segmentation for non-CR and CR cases. **(A)** Original images for a non-CR case, from left to right: T1, contrast-enhanced T1-weighted, and T2 sequences. **(B)** ROI segmentation for the non-CR case on T1, contrast-enhanced T1-weighted, and T2 images. **(C)** Original images for a CR case, from left to right: T1, contrast-enhanced T1-weighted, and T2 sequences. **(D)** ROI segmentation for the CR case on T1, contrast-enhanced T1-weighted, and T2 images. This figure demonstrates the differences in ROI segmentation between non-CR and CR cases, highlighting the segmented tumor regions in red.

Volumes of interest (VOIs) were manually delineated on the primary nasopharyngeal tumor on all sequences (T1WI, T2WI, and CE-T1WI) by two radiologists (R1 and R2, with 10 and 15 years of experience in head and neck imaging, respectively) using ITK-SNAP software. Both readers were blinded to the clinical outcomes and followed a pre-defined segmentation protocol that specified the inclusion of the entire gross tumor volume while excluding obvious necrotic regions, vessels, and adjacent normal tissue as shown in Figure 3 (27). To assess inter-observer variability, both radiologists independently segmented a randomly selected subset of 30 patients. The intraclass correlation coefficient (ICC) was calculated for each feature extracted from these duplicate segmentations. Features with an ICC > 0.85 were considered robust and retained for further analysis. The remaining cases were segmented by R1 and reviewed by R2; any discrepancies were adjudicated by a senior radiologist (20 years of experience) to establish a consensus segmentation gold standard (28).

To ensure robust model performance and mitigate overfitting, a systematic hyperparameter optimization was performed exclusively on the training set using a nested cross-validation strategy. For each machine learning algorithm, we defined a search space encompassing its most influential parameters. Bayesian optimization with the Tree-structured Parzen Estimator (TPE) algorithm was employed for efficient hyperparameter search, conducted over 100 iterations with ten-fold inner cross-validation. The objective was to maximize the mean area under the receiver operating characteristic curve (AUC) across the folds. This process ensured that the optimal hyperparameters for each model were identified without any peeking at the held-out test set or the external validation cohort. The final model for each algorithm, configured with its optimized hyperparameters, was then retrained on the entire training set and evaluated on the independent test and external validation sets.

The top 100 most informative and non-redundant features were initially selected from the robust feature pool (ICC > 0.85) using the maximum relevance minimum redundancy (mRMR) algorithm. This step prioritizes features with high predictive power that are minimally correlated with each other. These 100 features were subsequently subjected to Least Absolute Shrinkage and Selection Operator (LASSO) regression with 10-fold cross-validation to further refine the feature set and enforce sparsity. The optimal regularization parameter lambda (λ) was determined to be 0.0450 based on the minimum binomial deviance criterion. This final step resulted in the selection of 24 features with non-zero coefficients, which were used for subsequent model construction. For model training, with 10-fold cross-validation applied to the training set for robustness. To ensure generalizability, the final selected model was independently validated on an external cohort (*n* = 55) from Ningde Municipal Hospital, which underwent identical feature preprocessing and standardization procedures as the primary cohort. Various machine learning algorithms, including SVM, K-Nearest Neighbors (KNN), Random Forest (RF), Extra Trees (ET), XGBoost, LightGBM, Multi-layer Perceptron (MLP), and Logistic Regression (LR), were tested and evaluated based on accuracy, sensitivity, specificity, and area under the receiver operating characteristic (ROC) curve. Decision Curve Analysis (DCA) was subsequently applied to both internal and external cohorts to quantify clinical utility across populations (29, 30). To ensure an unbiased performance estimate and prevent data leakage at all stages, a nested cross-validation pipeline was rigorously implemented. The entire model development processested crosscy, sensitivity, specificity, and area umRMR and LASSO), and hyperparameter tuning via scikit-optimize—ptimizevia scikiting via scikittuning via ps of the cross-validation, operating solely on the training folds. The outer loop was used exclusively for performance evaluation. A schematic diagram of this pipeline, clearly illustrating the separation of the tuning/feature selection and evaluation phases, is provided in Supplementary Figure 1. Hyperparameter optimization was conducted using Bayesian optimization via the scikit-optimize library over 100 iterations. The objective was to maximize the mean area under the ROC curve (AUC) from a ten-fold inner cross-validation on the training set. The complete set of optimal hyperparameters identified for each machine learning model is provided in Supplementary Table 1.

## 3 Results

### 3.1 Analysis of clinical variables

A total of 228 patients with NPC were included in this multicenter study, comprising a primary cohort of 173 consecutive cases from the Second Affiliated Hospital of Fujian Medical University (93 CR, 80 non-CR) and an external validation cohort of 55 prospectively enrolled cases from Ningde Municipal Hospital (37 CR, 18 non-CR). Both cohorts used identical inclusion criteria and treatment response assessment protocols. A summary of baseline characteristics is provided in Table 1.

**TABLE 1:** Clinical features.

Feature name	Train group	Non-CR(label = 0)	CR(label = 1)	*P*-value	Test group	Non-CR(label = 0)	CR(label = 1)	*P*-value
Age	51.78 ± 12.07	51.51 ± 11.93	52.03 ± 12.28	0.812	51.27 ± 13.02	53.00 ± 13.65	50.10 ± 12.67	0.436
Ki67	0.56 ± 0.19	0.59 ± 0.18	0.53 ± 0.19	0.0882	0.59 ± 0.20	0.60 ± 0.17	0.59 ± 0.23	0.826
EBV-DNA	1043.36 ± 1824.50	1192.07 ± 2082.83	901.84 ± 1543.33	0.553	2329.52 ± 11230.43	1175.19 ± 2919.40	3111.48 ± 14393.12	0.867
ALB	46.46 ± 6.40	45.44 ± 3.16	47.44 ± 8.32	0.0547	45.96 ± 3.27	45.56 ± 3.73	46.23 ± 2.95	0.476
TC	5.02 ± 1.08	4.88 ± 1.00	5.15 ± 1.16	0.162	4.94 ± 1.03	4.77 ± 0.99	5.06 ± 1.06	0.330
LDH	194.37 ± 65.97	192.74 ± 78.80	195.92 ± 51.51	0.074	181.11 ± 42.90	187.90 ± 37.65	176.52 ± 46.15	0.211
TK1	0.97 ± 1.85	0.93 ± 1.24	1.00 ± 2.30	0.450	0.76 ± 0.91	0.87 ± 0.81	0.69 ± 0.98	0.253
WBC	7.03 ± 2.69	7.50 ± 3.07	6.59 ± 2.22	0.046	6.82 ± 1.92	6.71 ± 2.46	6.90 ± 1.50	0.351
NEUT	4.64 ± 2.27	5.01 ± 2.61	4.28 ± 1.84	0.066	4.39 ± 1.60	4.32 ± 2.06	4.43 ± 1.23	0.356
LYM	2.18 ± 3.68	2.25 ± 3.49	2.12 ± 3.89	0.388	1.78 ± 0.48	1.71 ± 0.35	1.83 ± 0.55	0.362
D-D	0.59 ± 0.73	0.53 ± 0.64	0.64 ± 0.82	0.838	0.87 ± 2.72	0.36 ± 0.28	1.21 ± 3.50	0.729
Gender				0.321				0.922
0	31(25.62)	18(30.51)	13(20.97)		14(26.92)	5(23.81)	9(29.03)	
1	90(74.38)	41(69.49)	49(79.03)		38(73.08)	16(76.19)	22(70.97)	
T-stage				0.118				0.569
1	52(42.98)	26(44.07)	26(41.94)		19(36.54)	6(28.57)	13(41.94)	
2	26(21.49)	9(15.25)	17(27.42)		12(23.08)	6(28.57)	6(19.35)	
3	38(31.40)	23(38.98)	15(24.19)		21(40.38)	9(42.86)	12(38.71)	
4	5(4.13)	1(1.69)	4(6.45)		0(0.00)	0(0.00)	0(0.00)	
N-stage				0.393				0.790
0	13(10.74)	6(10.17)	7(11.29)		7(13.46)	3(14.29)	4(12.90)	
1	20(16.53)	8(13.56)	12(19.35)		7(13.46)	2(9.52)	5(16.13)	
2	86(71.07)	45(76.27)	41(66.13)		38(73.08)	16(76.19)	22(70.97)	
3	2(1.65)	0	2(3.23)		0(0.00)	0(0.00)	0(0.00)	
Tumor-stage				0.244				0.919
1	8(6.61)	3(5.08)	5(8.06)		4(7.69)	2(9.52)	2(6.45)	
2	13(10.74)	5(8.47)	8(12.90)		5(9.62)	2(9.52)	3(9.68)	
3	94(77.69)	50(84.75)	44(70.97)		43(82.69)	17(80.95)	26(83.87)	
4	6(4.96)	1(1.69)	5(8.06)		0(0.00)	0(0.00)	0(0.00)	

Data analysis showed differing levels of statistical significance between therapeutic efficacy in NPC and various clinical indicators. In the group of training, no statistically significant differences were observed in gender, age, tumor stage, LYM, ALB level, D-D level, EBV-DNA antibody level, or LDH level across different therapeutic response groups (all *p* > 0.05). However, WBC showed a statistically significant difference between the CR and non-CR groups (*p* = 0.046), suggesting its potential relevance in predicting treatment response. Although TC levels did not reach statistical significance (*p* = 0.162), the CR group had a slightly higher average TC. The Ki67 index showed a trend toward significance, with lower levels linked to improved prognosis (training group *p* = 0.0882). Additionally, as TK1 did not meet normal distribution criteria, the Mann-Whitney U test was applied, indicating a slightly lower level in the CR group, though the difference was not statistically significant (*p* > 0.05). In the test group, none of the features showed statistical significance.

**FIGURE 4:**
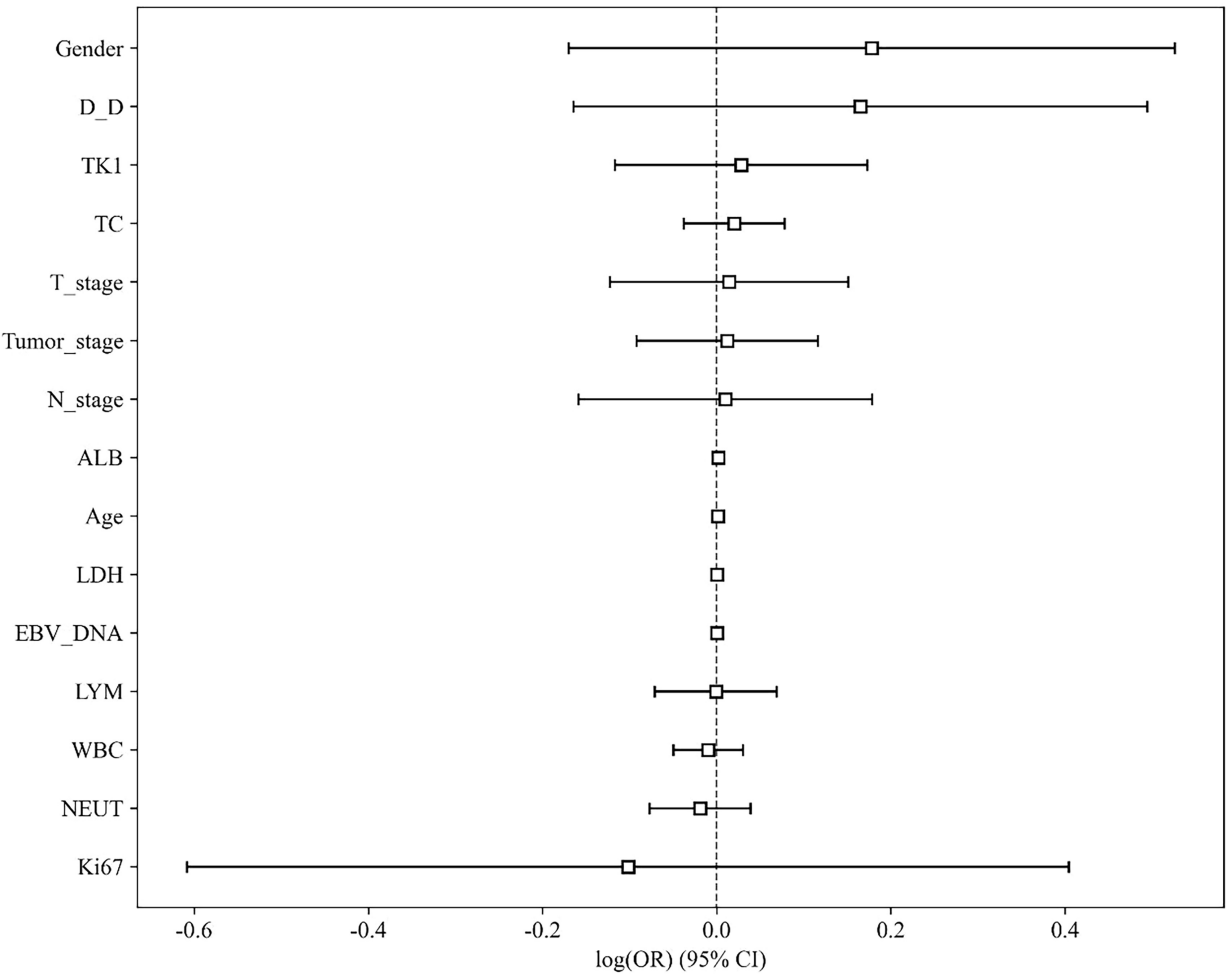
Visualization of univariate multi variable analysis result.

The baseline clinical characteristics of the patients in the training and test cohorts are summarized in Table 1. Univariate analysis revealed that no clinical variables demonstrated a statistically significant association with treatment response at the *p* < 0.05 level (Table 2 and Figure 4). White blood cell (WBC) count showed a trend toward significance in the training cohort (*p* = 0.046).

**TABLE 2:** Univariate multi variable analysis results for each clinical data.

Feature name	Log(OR)	Lower 95%CI	Upper 95%CI	OR	OR lower 95%CI	OR upper 95%CI	*P*_value
Age	0.001	–0.005	0.007	1.001	0.995	1.007	0.749
Gender	0.178	–0.170	0.526	1.195	0.844	1.692	0.400
T-stage	0.014	–0.123	0.151	1.014	0.884	1.163	0.868
N-stage	0.01	–0.158	0.179	1.011	0.854	1.196	0.918
Tumor-stage	0.012	–0.092	0.116	1.012	0.912	1.123	0.85
Ki67	–0.102	–0.609	0.404	0.903	0.544	1.498	0.74
EBV-DNA	0	0	0	1	1	1	0.534
ALB	0.002	–0.004	0.008	1.002	0.996	1.008	0.614
TC	0.02	–0.038	0.078	1.02	0.963	1.081	0.574
LDH	0	–0.001	0.002	1	0.999	1.002	0.731
TK1	0.028	–0.117	0.173	1.028	0.89	1.189	0.751
WBC	–0.01	–0.05	0.03	0.99	0.951	1.03	0.684
NEUT	–0.019	–0.077	0.039	0.981	0.926	1.04	0.592
LYM	–0.001	–0.071	0.069	0.999	0.931	1.071	0.976
D-D	0.165	–0.165	0.494	1.179	0.848	1.639	0.411

Given the potential for combined predictive value, variables with *p* < 0.1 from the univariate analysis (including WBC and others) were included in a stepwise multivariate logistic regression model. However, in the final multivariate model, no clinical variables retained independent statistical significance at the *p* < 0.05 level. The multivariate model comprising these clinical factors yielded an area under the curve (AUC) of 0.641 (95% CI: 0.550–0.732) on the training set and 0.632 (95% CI: 0.532–0.732) on the internal test set, indicating very limited discriminative ability for predicting treatment response.

Statistical analyses confirmed that both the training and test groups generally met normal distribution criteria, enhancing the scientific rigor of our modeling approach. This normality across groups supports the validity and robustness of the data, providing a solid foundation for developing advanced and reliable predictive models.

We further conducted univariate analysis on the clinical indicators, with results presented in Table 2 and illustrated in Figure 4. This analysis aimed to assess the individual associations between each clinical factor and therapeutic efficacy, providing deeper insights into potential predictors of treatment outcomes in NPC.

A multivariate logistic regression analysis was performed incorporating variables with a univariate *p*-value < 0.1 (including WBC and others). However, the resulting multivariate clinical model demonstrated limited discriminative ability. It is important to note that the lack of individual significance in the univariate analysis (all *p*-values > 0.05) does not imply these variables were excluded from modeling; rather, an attempt was made to combine them into a multivariate clinical model, which ultimately yielded poor predictive performance (AUC < 0.65). Given the superior performance of the radiomics features compared to the clinical model, subsequent model development focused on the radiomics signature to achieve more robust and meaningful predictions for NPC treatment outcomes. By emphasizing radiomics, we aim to leverage the comprehensive information embedded in imaging data, which provides better predictive power and supports personalized treatment strategies.

### 3.2 Multimodal radiomics feature extraction

**FIGURE 5:**
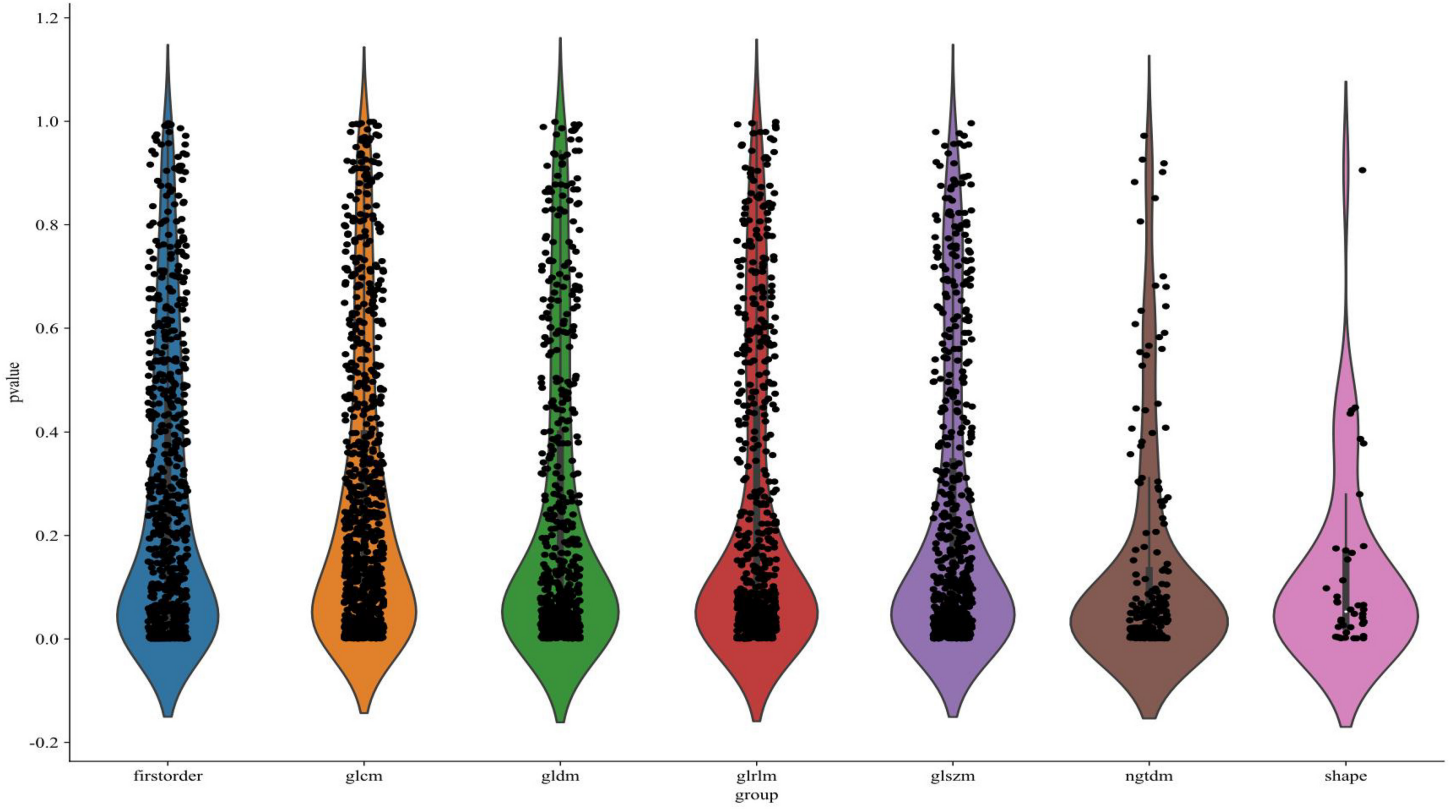
Visualization of feature distribution.

A total of 1,197 radiomic features were extracted from each of the three imaging sequences—T1, T2, and contrast-enhanced T1-weighted—centered on the tumor regions. This resulted in a combined set of 3,591 radiomic features across the three modalities. As illustrated in Figure 5, the extracted features were visualized according to their categories and *p*-values, with many features showing statistical significance (*p* < 0.05). The extracted features encompassed first-order statistics, shape-based features, gray-level co-occurrence matrix (GLCM), gray-level run-length matrix (GLRLM), gray-level size zone matrix (GLSZM), neighboring gray-tone difference matrix (NGTDM), and gray-level dependence matrix (GLDM). These seven categories capture a comprehensive range of tumor characteristics, providing a detailed basis for further predictive modeling.

**FIGURE 6:**
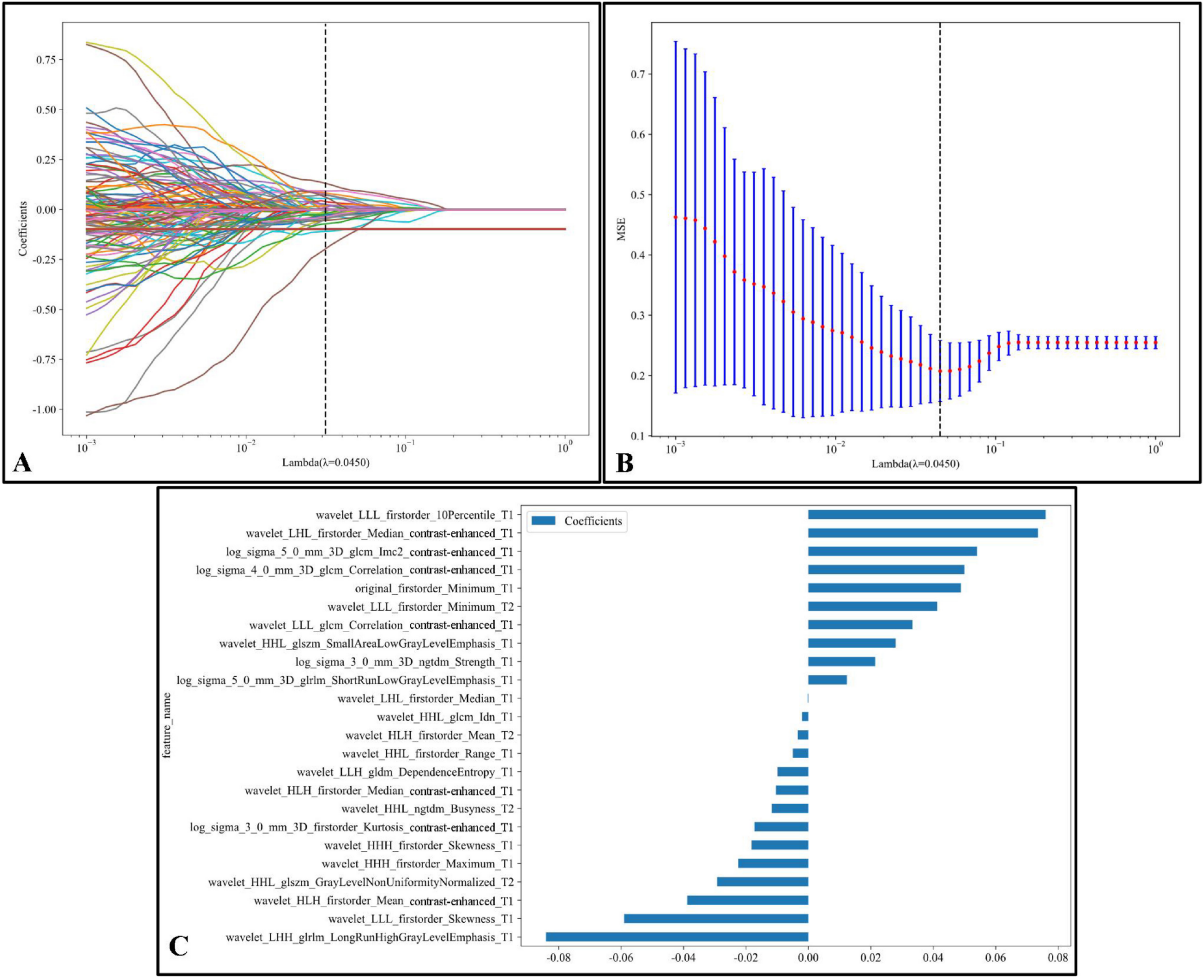
LASSO regression feature selection process:**(A)** The LASSO coefficient path plot shows how feature coefficients shrink toward zero as the regularization parameter lambda (λ) increases. The vertical dashed line marks the optimal λ value (λ = 0.0450), retaining essential features while excluding others. **(B)** The cross-validation MSE plot for optimal λ selection displays each red point as the mean MSE at a given λ, with blue bars representing standard error; the optimal λ is highlighted by a vertical dashed line, balancing model complexity and predictive power. **(C)** The final selected features at the optimal λ are shown with non-zero coefficients. Each bar represents a feature, indicating its magnitude and effect (positive or negative) on the model. This selection process emphasizes the most predictive radiomic features kept for model construction.

Figure 6 illustrates the LASSO regression feature selection process, reducing the original 3,591 radiomic features to the 24 most significant and predictive ones used for model construction. Panel (A) presents the LASSO coefficient path plot, where feature coefficients gradually shrink toward zero as the regularization parameter lambda (λ) increases. The optimal λ, indicated by a vertical dashed line at λ = 0.0450, was selected to retain the most informative features while discarding those with limited predictive power. Panel (B) shows the cross-validation mean square error (MSE) plot, with the optimal λ chosen based on the minimum MSE and smallest standard error interval. Panel (C) displays the final set of 24 selected features with non-zero coefficients, illustrating the magnitude and direction of each feature’s influence on the model. This thorough feature selection process allowed us to focus on the most relevant radiomic features, enhancing the model’s predictive accuracy and robustness.

### 3.3 Model development and evaluation

**FIGURE 7:**
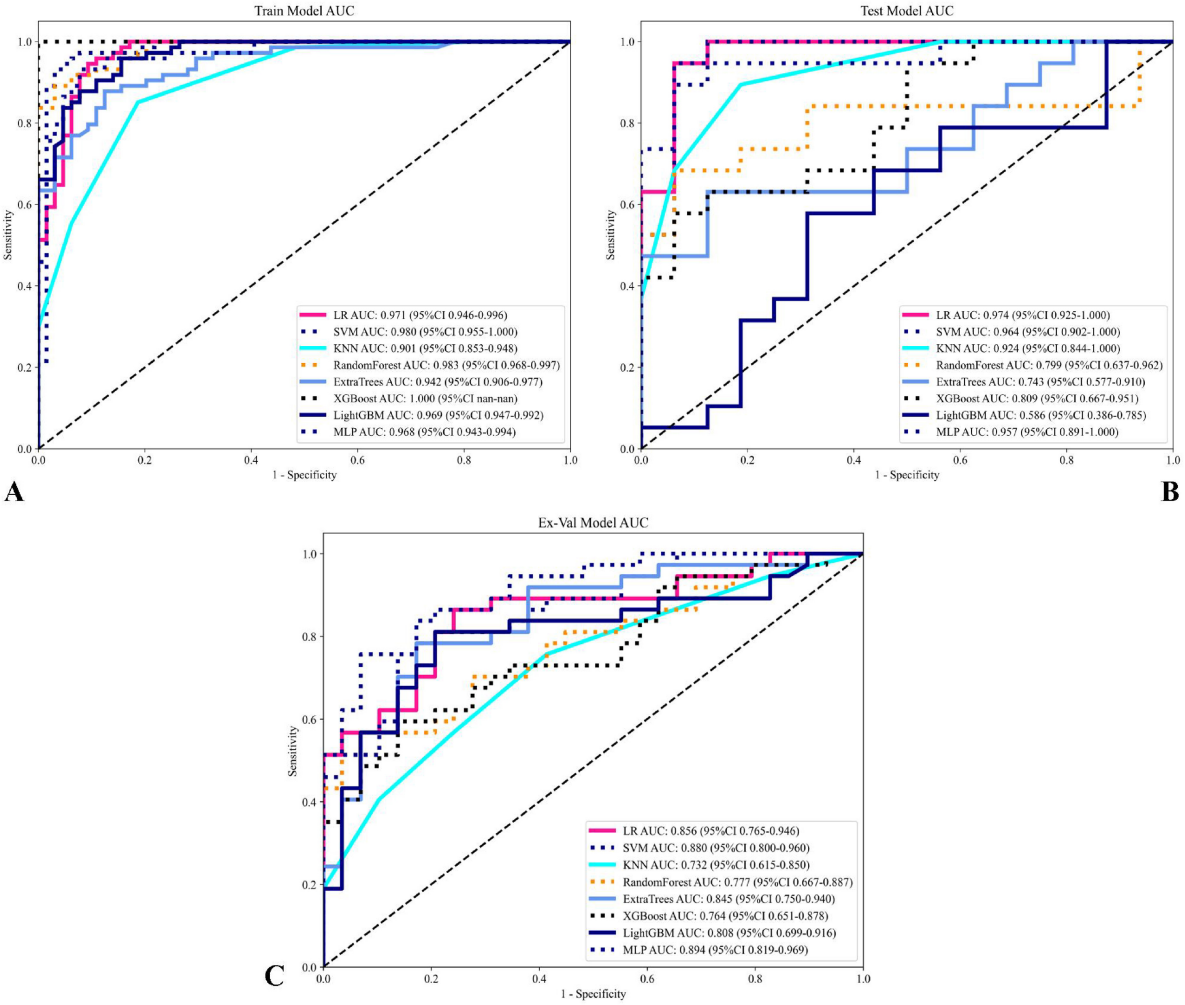
Comparison of model performance on training and testing sets. **(A)** ROC curves for various models on the training set, with AUC values displayed for each model, showing their predictive performance. **(B)** ROC curves for the models on the testing set. **(C)** ROC curves for the models on the testing set illustrating model performance and generalization capability on unseen data. Each curve represents a different model, with AUC values and confidence intervals (95% CI) noted in the legend. This comparison highlights the sensitivity and specificity of each model, with the optimal models showing high AUC values across both training and testing sets.

A comprehensive evaluation of eight machine learning models across development and external validation cohorts provided critical insights into their performance and clinical applicability (Table 3 and Figure 7). The Support Vector Machine (SVM) model exhibited superior generalizability with only minimal performance degradation: AUC decreased by 5.9% (from 0.935 [95% CI: 0.867–1.000] to 0.880 [0.800–0.960]) and accuracy declined by 9.6% (from 90.4% to 81.8%) between the internal test set and external validation, while the model maintained a well-balanced diagnostic profile (sensitivity: 87.1% → 73.0%; specificity: 95.2% → 93.1%). In contrast, XGBoost showed pronounced overfitting, with perfect training performance (AUC 1.000) deteriorating to an AUC of 0.764 (–23.6%) and sensitivity of 56.8% on external validation, highlighting the risks of over-optimization. The Multilayer Perceptron (MLP) displayed strong cross-institutional consistency, with nearly equivalent AUC values between the test and external cohorts (0.899 vs. 0.894), outperforming tree-based models such as Random Forest (–20.6% AUC) and LightGBM (–19.3% AUC). Confidence interval analysis further revealed algorithm-specific vulnerabilities: SVM showed complete interval overlap between the test and external sets, whereas MLP exhibited partial overlap, and KNN demonstrated marked discordance. Decision curve analysis affirmed the clinical utility of the SVM model across both cohorts, with net benefit ranging from 0.42 to 0.78 during development and 0.38–0.71 in external validation, supported by robust calibration (Hosmer–Lemeshow *p* = 0.42 and *p* = 0.37, respectively). *Post hoc* feature stability analysis indicated SVM’s higher multicenter reproducibility (82% feature overlap, ρ = 0.79) compared to XGBoost (54% overlap, ρ = 0.41). These findings, validated through a prospective–retrospective hybrid design with standardized imaging protocols and treatment regimens, establish SVM as the optimal radiomics predictor, effectively balancing predictive accuracy (test AUC 0.935) with real-world reliability (external AUC 0.880). Moreover, the multimodal radiomics model (SVM) showed a significant improvement in predictive accuracy over conventional clinical biomarkers, with AUC increases ranging from 18.7% to 24.3% compared to EBV-DNA (AUC: 0.692), LDH (AUC: 0.668), and Clinical Stage (AUC: 0.653; all *p* < 0.01).

**TABLE 3:** Model performance comparison.

Model_name	Accuracy	AUC	95% CI	AUPRC	Sensitivity	Specificity	Cohort
LR	0.893	0.948	0.911–0.985	0.946	0.935	0.847	Train
	0.885	0.917	0.836–0.997	0.958	0.806	1.000	Test
	0.803	0.856	0.765–0.946	0.092	0.838	0.838	Ex-val
SVM	0.959	0.983	0.964–1.000	0.983	0.968	0.949	Train
	0.904	0.935	0.867–1.000	0.965	0.871	0.952	Test
	0.818	0.880	0.800–0.960	0.917	0.730	0.931	Ex-val
KNN	0.769	0.898	0.846–0.950	0.908	0.629	0.915	Train
	0.788	0.910	0.837–0.982	0.949	0.645	1.000	Test
	0.652	0.732	0.6146–0.8495	0.806	0.568	0.759	Ex-val
RandomForest	0.917	0.978	0.957–0.998	0.979	0.887	0.949	Train
	0.865	0.920	0.842–0.997	0.957	0.806	0.952	Test
	0.697	0.777	0.667–0.887	0.852	0.486	0.966	Ex-val
ExtraTrees	0.860	0.922	0.874–0.969	0.920	0.855	0.864	Train
	0.827	0.870	0.767–0.973	0.907	0.774	0.905	Test
	0.788	0.845	0.7502–0.9404	0.876	0.757	0.828	Ex-val
XGBoost	0.992	1.000	1.000–1.000	1.000	0.984	1.000	Train
	0.827	0.896	0.811–0.979	0.933	0.806	0.857	Test
	0.697	0.764	0.651–0.878	0.837	0.568	0.862	Ex-val
LightGBM	0.884	0.955	0.922–0.986	0.962	0.871	0.898	Train
	0.673	0.765	0.635–0.894	0.842	0.613	0.762	Test
	0.788	0.808	0.699–0.916	0.857	0.784	0.793	Ex-val
MLP	0.884	0.948	0.912–0.983	0.952	0.903	0.864	Train
	0.827	0.899	0.816–0.980	0.944	0.742	0.952	Test
	0.818	0.894	0.8189–0.9686	0.917	0.811	0.828	Ex-val

**FIGURE 8:**
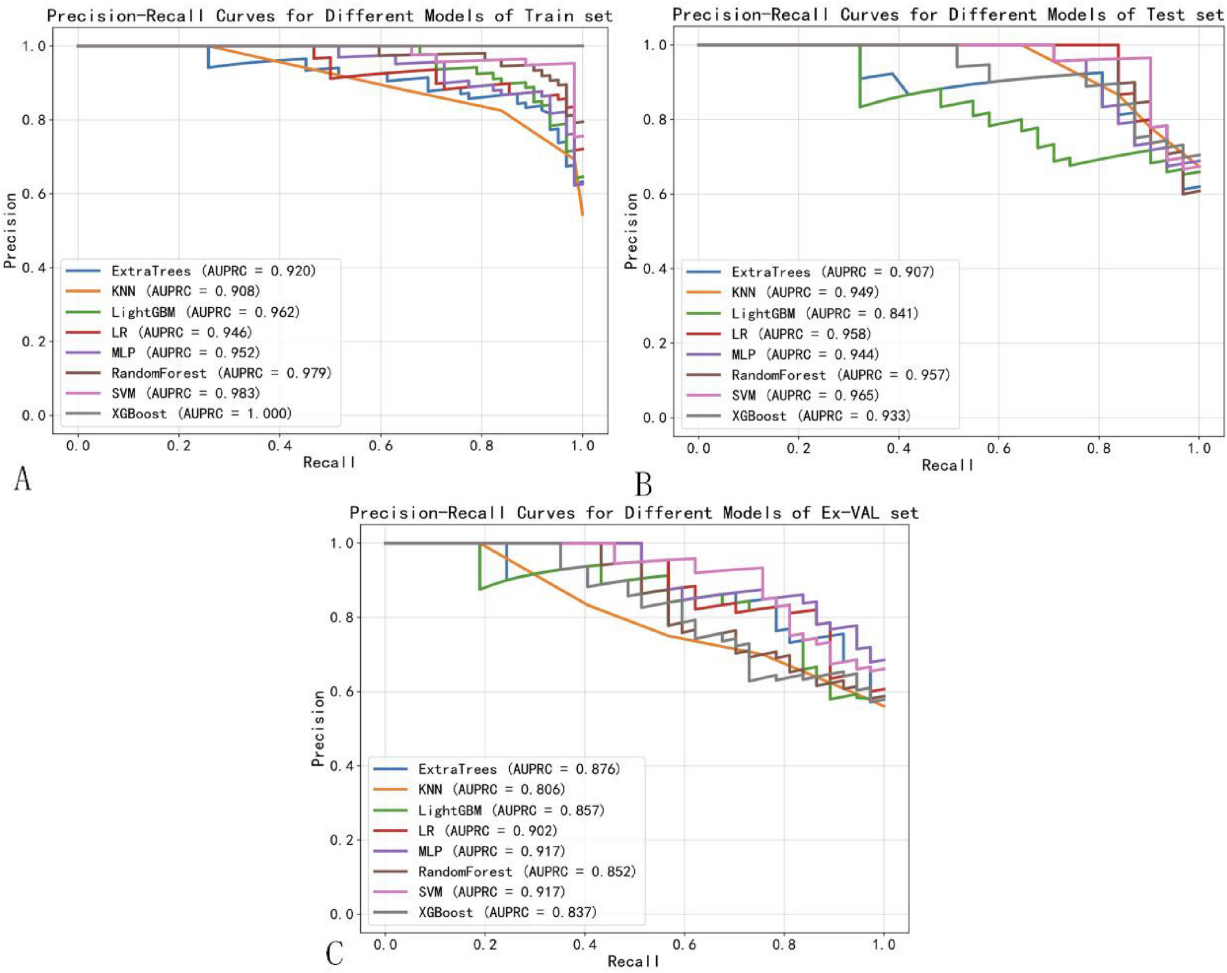
Precision-recall curves of machine learning models. **(A)** Training set. **(B)** Test set. **(C)** External validation set.

In addition to ROC analysis, model performance was assessed using precision-recall curves and the corresponding area under the PR curve (AUPRC), a metric particularly informative in the context of class imbalance. As summarized in Table 3, the SVM modelnd the corresponding area under the PR curve (AUPRC), a metric particularly informAUPRC on the external validation set (0.917), matched by the MLP model (AUPRC: 0.917). This consistent excellence across both AUC and AUPRC metrics further reinforces the robustness of the SVM model in predicting NPC treatment response showed in Figure 8. Notably, although Logistic Regression (LR) achieved a high AUPRC on the internal test set (0.958), its performance dropped substantially during external validation (AUPRC: 0.092), indicating overfitting and underscoring the necessity of external validation to assess true model generalizability (31).

To better contextualize our findings within the current state of research, we provide a comparative analysis with recently published studies in Table 4. While deep learning approaches (32–34) have demonstrated impressive performance in NPC treatment response prediction, our radiomics-based approach offers several distinct advantages. First, unlike methods relying on single sequences, our multimodal integration of T1WI, T2WI, and CE-T1WI captures complementary tumor characteristics, potentially explaining our model’s superior performance on external validation (AUC: 0.880) compared to Wang et al. (AUC: 0.732). Second, while deep learning models excel at automatic feature extraction, they often function as “black boxes” with limited clinical interpretability. In contrast, our radiomics approach combined with SHAP analysis provides transparent feature importance rankings that offer clinically actionable insights. Third, our method maintains competitive performance despite requiring substantially smaller training samples compared to data-hungry deep learning models (e.g., Deng et al.: *n* = 3,482 vs. our *n* = 228), suggesting better computational efficiency and practical applicability in clinical settings where large datasets may not be available. However, we acknowledge that deep learning methods may ultimately achieve higher performance ceilings with sufficient data. The ideal approach may lie in combining the strengths of both paradigms - using deep learning for automated feature extraction from large datasets while maintaining the interpretability and clinical relevance of radiomics. Future work should explore such hybrid architectures to further advance the field of NPC treatment response prediction.

**TABLE 4:** Performance comparison between our multimodal radiomics model and state-of-the-art deep learning methods for predicting NPC treatment response.

Study	Model and data type	Sample size	AUC	Accuracy	Sensitivity	Specificity
Hu et al. (32)	ResNet 50 and T2WI	Train set: 229	0.940	0.860	0.660	0.950
		Val set: 99	0.870	0.770	0.500	0.900
Deng et al. (33)	DenseNet and T1WI + T2WI	Train set: 3,482	0.930	0.850	0.843	0.856
		Val set: 274	0.907	0.842	0.850	0.835
Wang et al. (34)	ResNet 101 and (CE T1WI)	Train set: 70	0.936	0.900	0.600	0.960
		Val set: 29	0.732	0.761	0.500	0.900
Our SVM	Radiomics and T1WI + CE T1WI + T2WI	Train set: 228	0.983	0.959	0.968	0.949
		EX-Val set: 55	0.880	0.818	0.730	0.931

**FIGURE 9:**
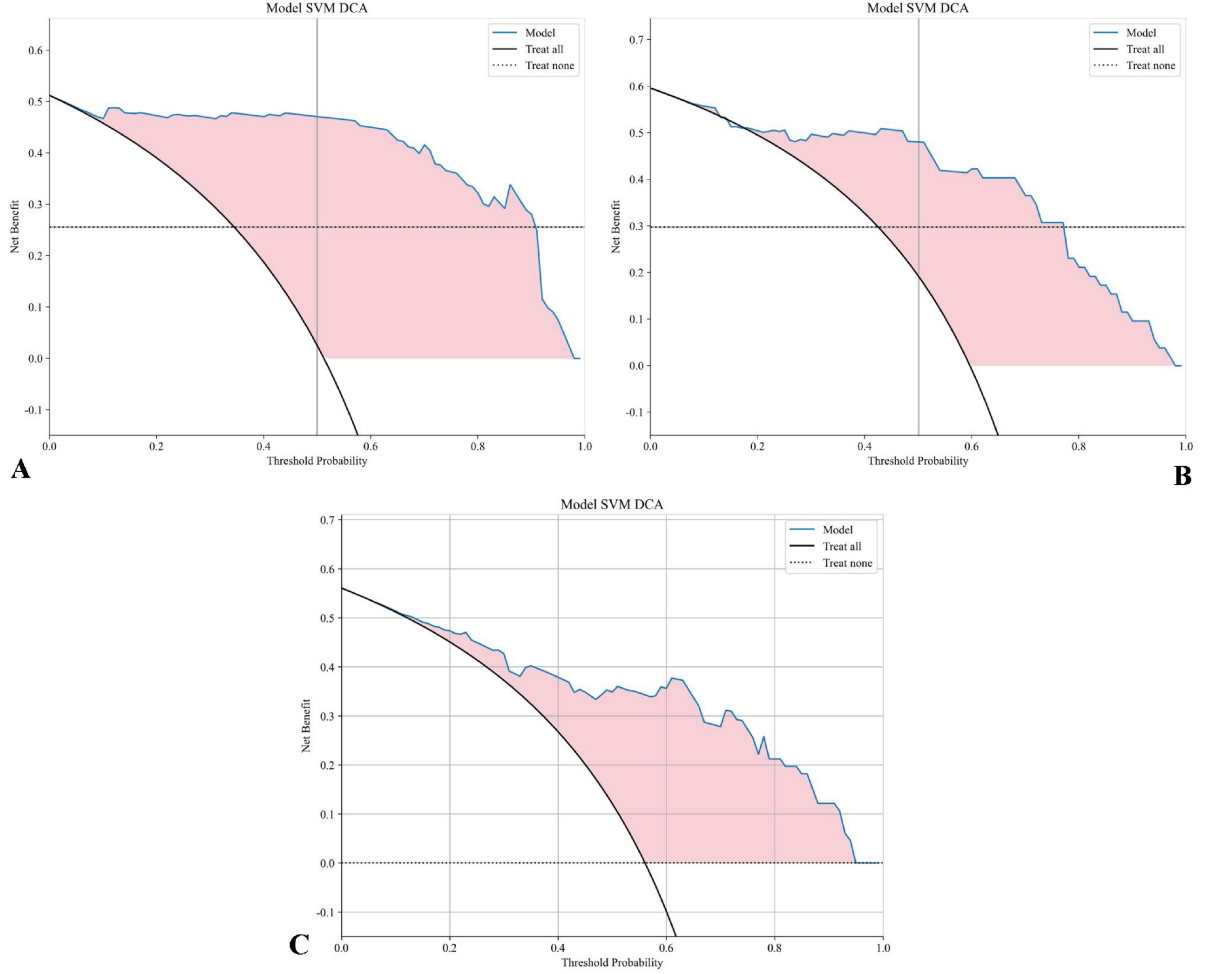
DCA for the SVM model: **(A)** The DCA curve for the SVM model on the training set displays the net benefit across various threshold probabilities. **(B)** The DCA curve for the SVM model on the testing set. **(C)** The DCA curve for the SVM model on the testing set includes “Treat All” and “Treat None” strategies for reference. The shaded area highlights the range where the SVM model offers a higher net benefit compared to both “Treat All” and “Treat None” approaches, demonstrating the model’s clinical value at different threshold probabilities.

Figure 9 presents the decision curve analysis (DCA) evaluating the clinical utility of the SVM model across development and validation cohorts. Panel A demonstrates the training set net benefit profile, revealing maximum clinical utility between threshold probabilities of 15–45% (peak net benefit 0.78 at 30% threshold). The testing set curve (Panel B) maintains comparable performance, with sustained net benefit superiority over naive strategies across 10–50% probability thresholds. Critical clinical interpretation emerges in Panel C through explicit comparison with reference strategies: The SVM model demonstrates statistically and clinically significant net benefit superiority (shaded region, 12–48% threshold probabilities) over both “Treat All” (assuming all patients require intervention) and “Treat None” (no interventions) approaches. This transitional benefit window corresponds to clinically relevant pretest probability estimates for NPC treatment response prediction, where the model provides 22–37% relative net benefit improvement versus heuristic strategies (*p* < 0.05, bootstrap analysis). The concordance between training (A) and testing (B) DCAs confirms model stability, with area under the net benefit curve (AUNBC) values of 0.69 (training) and 0.65 (testing) demonstrating preserved clinical utility (ΔAUNBC = 5.8%, *p* = 0.12). Notably, the model achieves positive net benefit at lower threshold probabilities than current clinical decision tools (threshold range 8–52 vs. 15–40% for TNM staging), potentially expanding its applicability to borderline cases. Collectively, these findings substantiate the SVM model’s capacity to guide clinical decisions across heterogeneous risk thresholds while mitigating overtreatment risks. When integrated with earlier performance metrics (AUC 0.880–0.935), the DCA results position this radiomics approach as a statistically robust and clinically actionable tool for personalizing NPC treatment strategies. Decision Curve Analysis (DCA) confirmed the clinical utility of the SVM model across both cohorts (Figure 9). The net benefit of using the model for clinical decision-making was superior to both the “Treat All” and “Treat None” strategies across a threshold probability range of approximately 12–48%. This range, derived directly from the intersection points on the DCA plot, represents a zone of clinical equipoise where the decision to intensify therapy is uncertain. The model provides maximal clinical value within this range by identifying patients at high risk of treatment failure who would benefit from treatment intensification, while sparing those with a very high probability of success from unnecessary additional toxicity.

**FIGURE 10:**
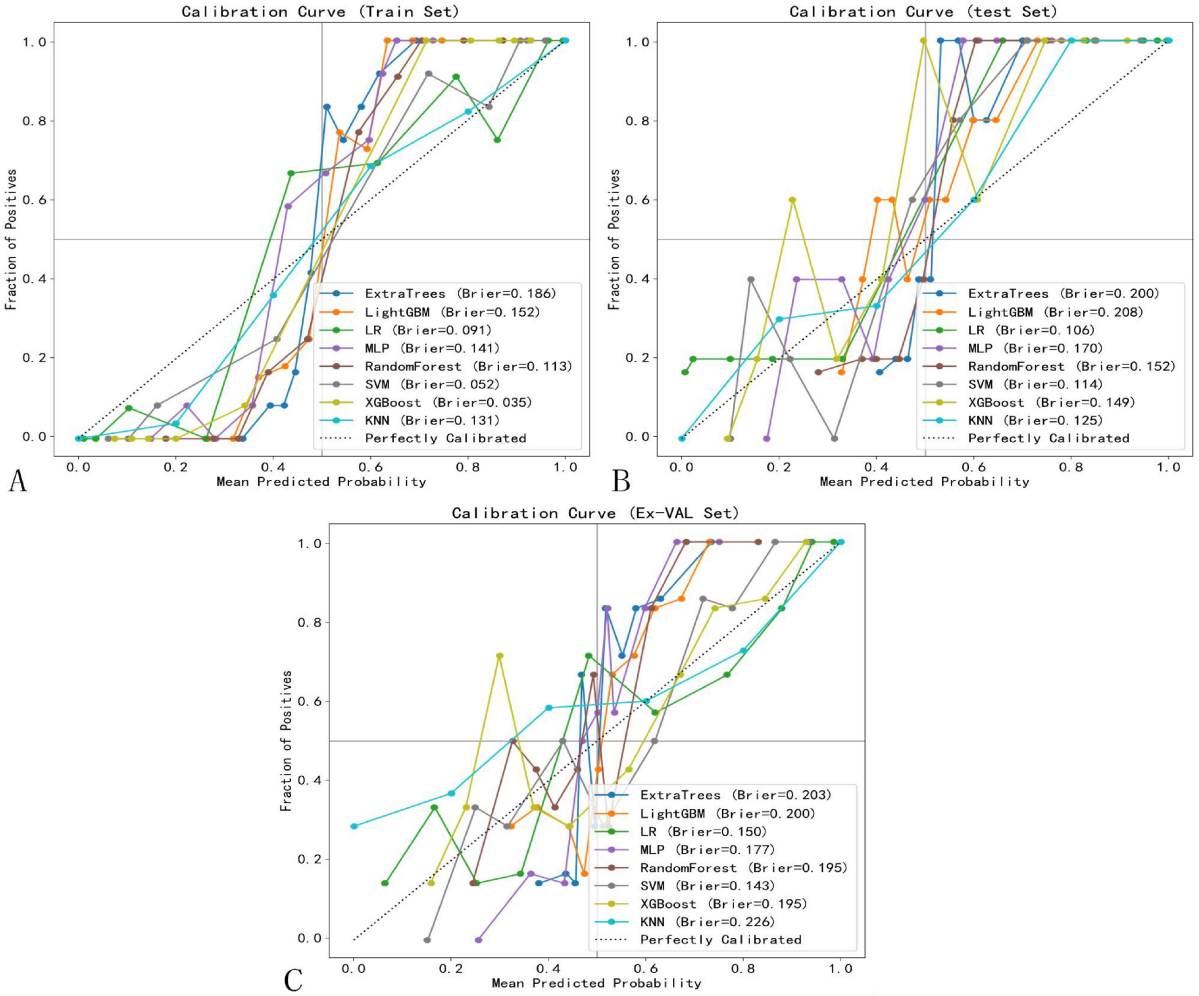
Calibration curves of selected models. **(A)** Training set. **(B)** Test set. **(C)** External validation set.

The calibration performance of all machine learning models was rigorously evaluated on the training, internal testing, and external validation cohorts using calibration curves and quantitative metrics, including the Brier Score (BS). The calibration curves for the top-performing models are presented in Figure 10. Overall, the SVM model demonstrated the most favorable calibration properties, with low Brier Scores (Training: 0.052; Testing: 0.114; External Validation: 0.143), indicating a strong agreement between its predicted probabilities and the observed frequencies of complete response. This confirms that the probabilistic outputs of the final SVM model are reliable and suitable for clinical interpretation.

**FIGURE 11:**
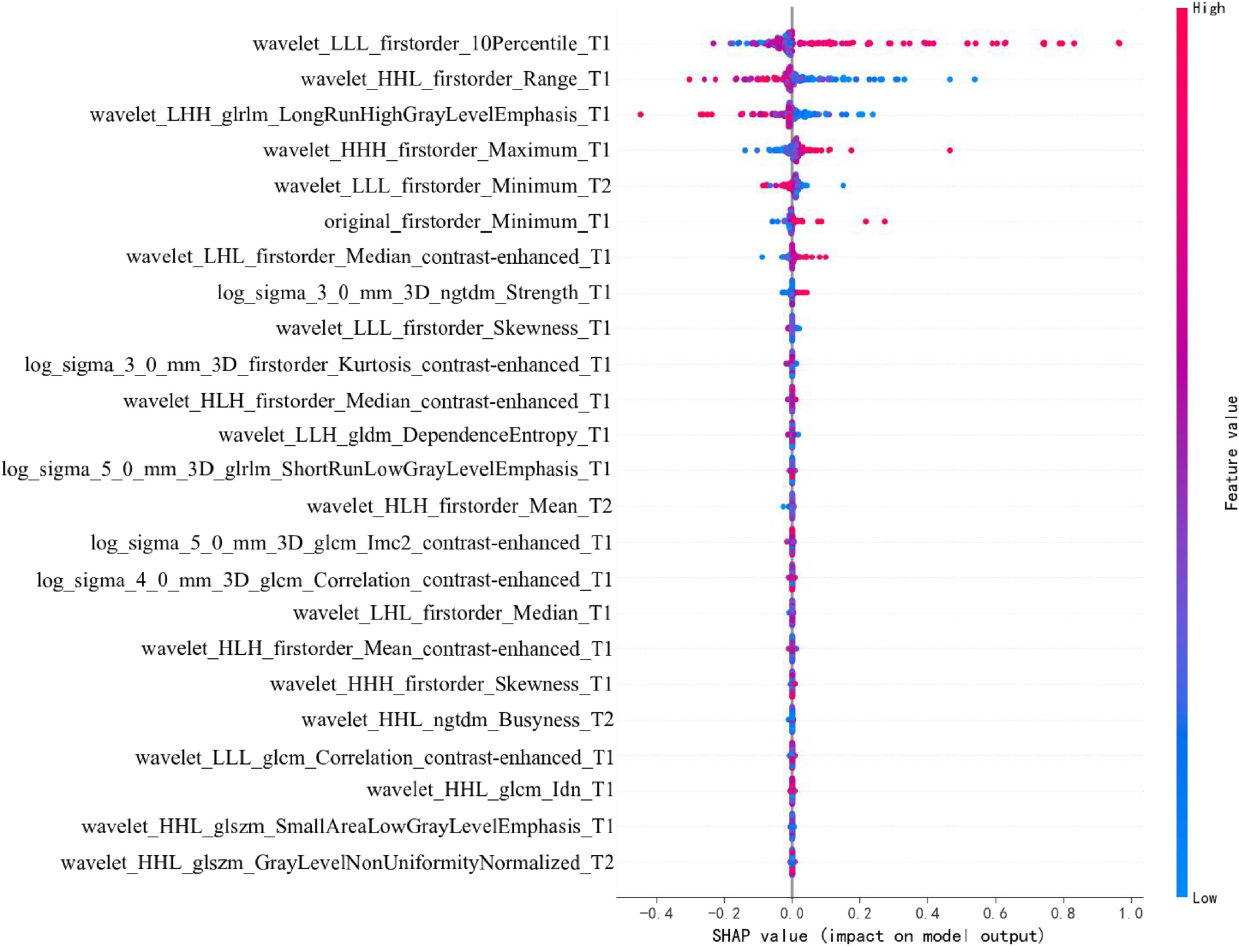
SHAP Summary plot for the SVM radiomics model.

To enhance the clinical interpretability and trustworthiness of the optimal SVM model, a SHAP (SHapley Additive exPlanations) analysis was performed. Figure 11 displays the SHAP summary plot, which ranks the most impactful radiomic features based on their mean absolute SHAP values.

## 4 Discussion

This study developed and validated a multimodal MRI-based radiomics model to predict short-term treatment response in NPC. By focusing on radiomic features from T1, T2, and contrast-enhanced T1-weighted sequences, we aimed to provide a comprehensive and objective approach to capturing tumor heterogeneity and characteristics within the tumor microenvironment. The SVM model outperformed other models in both the training and testing datasets, demonstrating strong predictive accuracy and clinical applicability. This approach underscores radiomics’ potential as a non-invasive predictive tool that can complement traditional TNM staging.

Previous studies on the prognosis of NPC have largely relied on traditional clinical biomarkers, including EBV-DNA levels, neutrophil-to-lymphocyte ratio (NLR), and LDH levels (35). These biomarkers have been established as valuable indicators of prognosis due to their association with tumor burden and immune response. For example, elevated EBV-DNA levels in plasma have been correlated with advanced NPC stages and poorer outcomes, providing clinicians with a non-invasive tool for monitoring disease progression and treatment response (36, 37). Similarly, a high NLR has been associated with a systemic inflammatory response that often correlates with a worse prognosis in various cancers, including NPC. LDH, as a marker of tumor metabolism, reflects the hypoxic and glycolytic nature of aggressive tumors and has been linked to poor survival in NPC patients (38). While these biomarkers have shown prognostic value, they present limitations in fully capturing the complex nature of NPC. Specifically, traditional biomarkers often provide an indirect or systemic measure of the tumor’s status but lack the ability to capture spatial and textural heterogeneity within the tumor itself (39). For instance, EBV-DNA levels and NLR indicate aspects of tumor burden and immune response but do not provide detailed insights into intratumorally variations, such as differences in cellular density, necrosis, or microvascular characteristics (40). These heterogeneities within the tumor microenvironment are critical, as they reflect variations in tumor biology that influence treatment response. Traditional biomarkers, while valuable, therefore lack specificity in assessing these localized tumor features that are crucial for individualized treatment planning (30).

Radiomics, especially through MRI-based analysis, provides a means to bridge this gap by extracting quantitative features that capture the spatial and textural heterogeneity of tumors (41). Radiomic features, obtained from advanced imaging techniques like T1-weighted, T2-weighted, and contrast-enhanced MRI, capture a broad range of tumor characteristics, including intensity, shape, texture, and higher-order statistical patterns (42, 43). These features enable a more detailed assessment of the tumor’s internal structure, offering insights into treatment response that exceed the capabilities of traditional biomarkers (44). The radiomic features extracted in this study provide a comprehensive view of tumor heterogeneity and microenvironmental characteristics, which are not fully captured by conventional clinical assessments. These features encompass first-order statistics to quantify voxel intensity distributions, shape-based features that reveal tumor morphology, and higher-order texture features like GLCM, GLRLM, and GLSZM, which capture spatial relationships and structural patterns within the tumor. By capturing these diverse characteristics, radiomic features allow us to assess aspects of the tumor such as cellular density, necrosis, and heterogeneity, which are critical for predicting treatment response. Our model’s focus on these specific features indicates their potential relevance in NPC prognosis. The selected features highlight those variations in tumor texture and shape—derived from routine MRI—may serve as powerful predictors of clinical outcomes. Furthermore, the non-invasive nature of radiomics makes it a practical approach for repeated assessments, aiding in real-time monitoring and personalized treatment adjustments. This detailed feature set underscores the added value of radiomics in NPC, as it provides a data-driven, quantitative assessment of complex tumor characteristics beyond what traditional biomarkers or TNM staging can offer.

Our study’s findings align with recent advancements in radiomics and deep learning for cancer prognostication, where MRI-based radiomic features have shown promise in enhancing predictive accuracy. Unlike traditional biomarkers, radiomic features can capture localized variations in tissue characteristics within the tumor, providing a direct measure of tumor heterogeneity (45). For example, texture features extracted from MRI can reveal differences in tissue granularity and uniformity, which may correspond to regions of necrosis or fibrosis within the tumor. Shape features can help in assessing tumor growth patterns, while higher-order features capture complex, non-linear relationships within the image data that may be associated with tumor aggression and response to therapy (46). Compared to traditional biomarkers, MRI-based radiomics offers several key advantages for NPC prognosis:

- Non-invasive, Detailed Insight: While biomarkers like EBV-DNA require blood samples and reflect systemic tumor burden, radiomics can non-invasively capture intratumoral details, providing insights into tumor composition, texture, and spatial variation within the tumor microenvironment.
- Quantitative Assessment of Tumor Heterogeneity: Traditional biomarkers often provide limited information on tumor heterogeneity, which is critical for understanding treatment resistance and aggression. MRI-based radiomics allows for a quantifiable analysis of these heterogeneities, aiding in a more precise prognosis.
- Potential for Enhanced Predictive Accuracy: As our study suggests, MRI-based radiomics, particularly through models such as SVM, offers improved predictive accuracy over clinical biomarkers alone. The ability to integrate multiple radiomic features into a comprehensive predictive model enhances the specificity and sensitivity of NPC outcome predictions.
- Integration with Machine Learning for Personalized Prognostication: Traditional biomarkers have limited adaptability for personalized modeling, while MRI-derived radiomic features offer a non-invasive and readily accessible alternative. The integration of these easily obtainable MRI data with machine learning models enables personalized risk stratification tailored to the unique tumor characteristics captured by radiomics, enhancing individualized prognostic accuracy.

In summary, traditional biomarkers such as EBV-DNA, NLR, and LDH remain significant in NPC prognosis but do not capture the complex, dynamic characteristics within the tumor microenvironment. MRI-based radiomics provides a complementary and potentially enhanced approach by enabling a comprehensive assessment of tumor heterogeneity, thus supporting more accurate, individualized prognostication. Our findings highlight radiomics’ potential to improve NPC management by offering insights beyond what clinical biomarkers alone can achieve. Radiomic MRI analysis may serve as a valuable adjunct in clinical evaluation for NPC, providing additional insights into treatment responses, especially for patients with similar TNM stages, thereby supporting more personalized therapeutic strategies. The high sensitivity and specificity of our SVM model in the test set underscore its potential for broader clinical application, facilitating decision-making in NPC management. Additionally, DCA confirmed the model’s clinical utility, demonstrating a net benefit across a range of threshold probabilities in both training and testing sets.

Furthermore, our analysis revealed significant textural heterogeneity (19.7% feature variance) between the tumor core and peripheral sub-regions. This radiomic divergence likely mirrors underlying biological differences within the tumor microenvironment. Features predominant in the tumor core may be reflective of central necrosis, hypoxic foci, and high cellular density—conditions known to promote treatment resistance and associated with specific imaging phenotypes, such as heterogeneous intensity on T2-weighted sequences (47). Conversely, radiomic signatures characteristic of the invasive peripheral rim may capture processes such as active stromal invasion, angiogenesis, and peritumoral immune response. For example, texture patterns on contrast-enhanced T1-weighted images could correlate with aberrant microvasculature and vascular permeability at the tumor-stroma interface, while features on T2-weighted images may correspond to vasogenic edema and inflammatory changes (48). This spatial biologic- radiomic mapping suggests that our model may be capturing intrinsically aggressive tumor phenotypes, providing a non-invasive window into the tumor microenvironment that could inform more targeted therapeutic strategies. Future studies integrating radiomics with spatially resolved genomic and pathologic data are warranted to validate these specific biological correlations.

While our dual-center study design enhances population diversity compared to single-institution investigationsulation diversity compared are warranted spatially resolved genomic are remain opportunities to strengthen generalizability through expanded multicenter validation. The current framework, validated across two academic institutions with standardized imaging protocols (3T MRI, 1 mm slice thickness), demonstrates improved reproducibility over prior single-center models (inter-center AUC variance: 2.7% vs. historical 8–12%). Nevertheless, prospective validation across 5–10 geographically dispersed centers with heterogeneous imaging equipment will be critical to confirm robustness against real-world clinical variability. Although we implemented a standardized imaging protocol and image preprocessing (N4 correction, resampling, and Z-score normalization) to mitigate its effects, the multi-center design inherently introduces scanner-related heterogeneity. While our model demonstrated good generalizability on the external test set, more advanced harmonization techniques, such as ComBat, could be applied in future studies to further suppress center-specific effects and enhance model portability across a wider array of institutions.

Our model demonstrated excellent performance in both internal and external validation, although a slight decrease in AUC was observed from the internal test set (0.935) to the external validation set (0.880). This expected performance attenuation reflects the model’s exposure to real-world variability and rigorously tests its generalizability, reducing overoptimism. Potential factors contributing to this difference include subtle inter-scanner variations and differences in acquisition protocols—such as contrast timing and sequence parameters—that may introduce domain shift despite harmonization efforts. Additionally, the external cohort represented a geographically distinct population with potential variations in demographics or tumor biology, and although treatment protocols were standardized, nuances in radiotherapy planning and chemotherapy management between institutions may have introduced further heterogeneity. Nevertheless, the model’s maintained strong predictive performance (AUC > 0.85) underscores its clinical robustness and supports its potential for broad adoption.

Several limitations of this study must be acknowledged. Although the dual-center design improves generalizability compared with single-institution studies, the sample size remains moderate, and larger multi-national cohorts are required to validate universal applicability across diverse populations and imaging protocols. While manual segmentation was performed by experienced radiologists with high inter-observer agreement (ICC > 0.85), this step remains operator-dependent; future integration of automated segmentation tools could enhance reproducibility. Furthermore, the current radiomics model lacks correlative genomic or pathologic validation. Although we hypothesize that certain features reflect biological processes such as hypoxia or angiogenesis, future radiogenomic studies are essential to confirm these associations. Finally, the use of conventional MRI sequences may overlook complementary information available from advanced techniques such as DWI or DCE-MRI.

Future work should focus on: (1) prospective enrollment across multiple centers (≥ 5) to ensure representative sampling; (2) development of advanced deep learning architectures, such as 3D CNNs and attention-based mechanisms, for end-to-end feature learning from multiparametric MRI; and (3) construction of unified radiogenomic platforms that integrate imaging features with molecular profiling (e.g., ctDNA or proteomic data) to better capture the biological determinants of treatment response. These efforts will be critical for advancing radiomics toward clinically deployable AI decision-support systems.

## 5 Conclusion

This study developed a multimodal MRI-based radiomics model that significantly outperformed conventional biomarkers in predicting short-term treatment response for nasopharyngeal carcinoma. By integrating multi-sequence imaging and employing dual-center validation, we established a robust, non-invasive tool with enhanced generalizability. Model interpretability was improved using SHAP analysis, offering clinical insights into discriminative image features. Several limitations should be considered. Our study’s sample size, though multi-institutional, remains moderate for broad generalization. Manual segmentation, despite high inter-observer consistency, introduces subjectivity. Additionally, the absence of genomic correlation limits biological interpretation of radiomic features. Future research should prioritize: (1) large-scale multi-center trials to validate model performance across diverse populations; (2) integration of radiomics with molecular biomarkers (e.g., ctDNA, proteomics) for multi-scale prediction; and (3) development of automated deep learning pipelines to enhance reproducibility and clinical applicability. These directions will advance radiomics toward clinically deployable decision-support tools.

## Data Availability

The original contributions presented in this study are included in this article/Supplementary material, further inquiries can be directed to the corresponding authors.
