# Supplementary material for: Multimodal MRI-based radiomics model for predicting short-term efficacy in nasopharyngeal carcinoma
Source: Front Med (Lausanne). 2025 Nov 21;12:1654023. doi: 10.3389/fmed.2025.1654023 (PMC12678374; doi:10.3389/fmed.2025.1654023)
Supplement: Supplementary file 1 [file Table_1.docx]

**Table S1. Machine Learning Model Hyperparameter Optimization Results**

| **Model** | **Hyperparameter** | **Description** | **Optimized Value** |
| --- | --- | --- | --- |
| **LR** | C | Regularization strength | 0.01 |
|  | penalty | Regularization type (L1/L2) | L2 |
|  | solver | Optimization algorithm | liblinear |
|  | max_iter | Maximum iterations | 100 |
| **SVM** | C | Regularization parameter | 0.1 |
|  | kernel | Kernel type | linear |
|  | gamma | Kernel coefficient | scale |
| **XGBoost** | learning_rate | Step size shrinkage | 0.01 |
|  | max_depth | Maximum tree depth | 10 |
|  | min_child_weight | Minimum sum of instance weight | 1 |
|  | n_estimators | Number of boosting rounds | 50 |
|  | reg_alpha | L1 regularization term | 0 |
|  | reg_lambda | L2 regularization term | 10 |
| **LightGBM** | learning_rate | Step size in boosting | 0.01 |
|  | max_depth | Maximum tree depth | 10 |
|  | num_leaves | Number of leaves in trees | 31 |
|  | n_estimators | Number of boosting iterations | 200 |
|  | reg_alpha | L1 regularization term | 0.1 |
|  | reg_lambda | L2 regularization term | 0.1 |
| **ExtraTrees** | n_estimators | Number of trees in forest | 200 |
|  | max_depth | Maximum depth of trees | 10 |
|  | min_samples_split | Minimum samples to split node | 10 |
|  | min_samples_leaf | Minimum samples at leaf node | 4 |
|  | max_features | Number of features for split | None |
| **Random Forest** | n_estimators | Number of trees in forest | 100 |
|  | max_depth | Maximum depth of trees | None |
|  | min_samples_split | Minimum samples to split node | 2 |
|  | min_samples_leaf | Minimum samples at leaf node | 1 |
|  | max_features | Number of features for split | sqrt |
|  | bootstrap | Whether use bootstrap samples | True |
| KNN | n_neighbors | Number of neighbors | 5 |
|  | weights | Weight function | uniform |
|  | metric | Distance metric | minkowski |
|  | p | Power parameter | 2 |
| **MLP** | hidden_layer_sizes | Neurons in hidden layers | (100,) |
|  | activation | Activation function | relu |
|  | solver | Weight optimization solver | adam |
|  | alpha | L2 regularization parameter | 0.0001 |
|  | learning_rate | Learning rate schedule | constant |
|  | learning_rate_init | Initial learning rate | 0.001 |
|  | max_iter | Maximum iterations | 200 |
|  | early_stopping | Whether use early stopping | False |

Training set

N=121

Test set

N=52

Test set

N=55

Feature Extraction

Feature Selection

(mRMR & LASSO)

Training model

(10-fold-Cross-validation)

Best model

Cross-validation performance of training set

Extract the selected features

Performance of Test set

Performance of Ex-Val

Figure S1. Nested Cross-Validation Pipeline for Radiomics Model Development and Validation
